# The effect of a dementia awareness class on changing dementia attitudes in adolescents

**DOI:** 10.1186/s12877-020-01589-6

**Published:** 2020-06-02

**Authors:** Nicolas Farina, Laura J. Hughes, Ellen Jones, Sahdia Parveen, Alys W. Griffiths, Kathleen Galvin, Sube Banerjee

**Affiliations:** 1 Centre for Dementia Studies, Brighton and Sussex Medical School, University of Sussex, Falmer, Brighton, BN1 9RY UK; 2 Brighton and Hove Dementia Action Alliance, Brighton, UK; 3 Centre for Applied Dementia Studies, University of Bradford, Bradford, UK; 4 Centre for Dementia Research, Leeds Beckett University, Leeds, UK; 5 School of Health Sciences, University of Brighton, Brighton, UK; 6 Faculty of Health, University of Plymouth, Plymouth, UK

**Keywords:** Young people, Dementia friends, Satisfaction, Stigma, Dementia, Awareness

## Abstract

**Background:**

Current evidence suggests that negative and stigmatising attitudes towards dementia may develop at a young age. There are a number of dementia education and awareness initiatives aimed at reducing stigma, though they have not been robustly evaluated to establish the impact on dementia attitudes or suitability in adolescent populations. This study explored the efficacy and satisfaction of one such initiative (Dementia Friends) in a British adolescent sample.

**Methods:**

301 adolescents (M = 12.6 years old, SD = 0.73) were assigned to either receive Dementia Friends (a 60-min interactive class that teaches about dementia and its effects on people’s lives) or education as usual. All participants completed a series of validated questionnaires pre- and post-intervention, related to dementia attitudes (Brief A-ADS and KIDS).

**Results:**

Adolescents in the dementia awareness group showed little to no improvements between time-points. The change scores in the dementia awareness group did not significantly differ to the control group based on both KIDS (d = − 0.003, *p* = 0.98) and Brief A-ADS (d = 0.14, *p* = 0.13) measures. There was no *Group x Time* effect after controlling for confounding variables.

**Conclusions:**

Dementia Friends is successful in terms of reach and impact, though this study suggests that it may fall short of achieving its goal of improving attitudes towards dementia. Importantly, Dementia Friends did not have a negative effect on attitudes, and the majority of adolescents enjoyed the sessions. It is important that these findings are replicated in a larger randomised-controlled study.

## Background

Stigma leads to social isolation, reduced quality of life and loss of independence for people with dementia [1]. It is also a major barrier for seeking and accessing support, diagnosis, treatment and information [2–4]. As such, reducing stigma towards people with dementia is a key policy priority worldwide [1, 5, 6] and features in the World Health Organisation Global Action Plan [7].

Negative attitudes and stigma towards dementia appear to already exist during adolescence [8, 9], however, some have reported that the stigmatic beliefs towards dementia are relatively low [10]. Irrespective, negative attitudes towards mental illness more broadly form early in life [11, 12]. Conceptually, education is seen as an important route to reduce stigma [13], and is effective at reducing stigma toward mental illness in adolescents [14]. Therefore, raising awareness of dementia in young people can be seen as an important method of reducing stigma. Embedding this within the education system would allow for a grand vision of the creation of a ‘Dementia Friendly Generation’.

There is a general lack of transparency surrounding the availability and uptake of dementia awareness initiatives. Within the UK, the Alzheimer’s Society has been one of the key driving forces of raising awareness of dementia through their Dementia Friends programme. Dementia Friends provides a one-off information session that provides people with a basic overview of dementia; it “*tackles the stigma and discrimination people with dementia can face globall*y” [15] (p. 1). In terms of numbers, the Dementia Friends initiative has been a great success being the UK’s “*biggest ever initiative to change people’s perceptions of dementia”* [15] (p. 1)*.* Like many other dementia friendly initiatives however, they generally have not been systematically or robustly evaluated [16, 17]; therefore, the reaction to it and the extent that it changes perceptions of dementia is unclear.

This is not to say that dementia awareness initiatives aimed at young people do not exist and have not been evaluated. One example being the Kids4dementia programme [18]. The Australian created programme is a teacher-led multimedia resource, that lasts 150-min over multiple sessions. The authors found a small to medium effect size on changing attitudes towards dementia following the programme, depending on whether the young people (aged 9–12) had previously heard of dementia before the programme. A number of dementia awareness initiatives have been developed with adolescents specifically in mind [19–22], though they have not yet been robustly evaluated using standardised and validated questionnaires. As a result, whilst dementia awareness initiatives should theoretically improve attitudes in adolescents, there is a lack of evidence to support this claim. Establishing whether such dementia awareness initiatives are effective in improving dementia attitudes in adolescents is essential before trying to implement these programmes on a wider scale.

Dementia Friends has been widely utilised by young people in the UK, with versions of the initiative being adapted for use within these age groups (e.g. 11–13 year olds). As of March 2019, there are over 291,000 young (aged 5–25 years old) Dementia Friends (Alzheimer’s Society, personal communication, 2019). Inherently, Dementia Friends can be seen as a positive initiative both in adults and young people. However, it is unclear whether these sessions are effective for adolescents in terms of changing attitudes towards dementia. A single study demonstrated that Dementia Friends was able to improve dementia knowledge and social comfort (factors from the Dementia Attitude Scale) in a group of US college students and community members in a pre- post-test design (*n* = 80) [23]. To our knowledge, there has been no peer-reviewed literature about the efficacy of the Dementia Friends initiative in adolescents.

At present, dementia awareness initiatives do not appear to occur widely within the UK education system, despite clusters of localised initiatives existing, and secondary school staff viewing it as an important topic [24]. Providing robust evidence that a dementia awareness initiative (i.e. Dementia Friends) is both meaningful to adolescents and able to improve attitudes towards dementia is important for schools to uptake the initiative. Such evidence is vital to allow resource and time-limited schools to make informed decisions about the value of implementation of dementia awareness initiatives within an already busy school curriculum.

The aim of this study was to understand the short-term effect of the Dementia Friends initiative on adolescents’ attitudes towards dementia whilst gaining some insight into their satisfaction of the session.

## Methods

### Participants and setting

Secondary schools in an area of England were approached for participation in this research. Schools were initially approached based on previous interest in hearing about dementia awareness initiatives, as part of a previous study [24]. Schools were approached using publically available information, there was no criteria for the order in which the schools were approached, though a preference was made for schools geographically close to Brighton and Hove. Senior members of staff (e.g. head teachers) provided consent for the research to occur within the schools. In recognition of the practicalities of working within a busy school environment, senior members of staff were able to select which classes (and pupils) were involved in the research. The only criteria were that the classes had to cater to adolescents aged 12–16.

Participants were recruited from three schools. All schools were mixed gender and rated ‘good’ according to Ofsted, a governmental organisation responsible for inspecting schools in England. None of the schools provided dementia education to their pupils. However, there were some variations between schools:

- School 1 is an academy sponsor led^1^ mainstream school with a Progress 8 score^2^ > 0.
- School 2 is a community school, with a Progress 8 score > 0.
- School 3 is a community school, with a Progress 8 score < 0.

Information about the schools were correct as of April 2019 [25].

The study was not preregistered in an independent, institutional registry. Ethical approval was obtained through the Brighton and Sussex Research Governance and Ethics Committee.

### Measures

The following measures were taken:

A. Demographics (e.g., age, gender, and ethnicity) (t1)
B. Adolescent Level of Contact of Dementia scale (ALoCDs)(t1, t2) – a validated measure of level of contact with people with dementia, designed for use in adolescents [26]. The scale provides scores for direct (i.e. ‘I have spent time with people with dementia’) and indirect (i.e. ‘I have watched TV shows or movies in which a character has dementia’) contact. It has shown adequate internal consistency (α = .89 and α = .62, respectively). Items are responded to on a 5-point Likert scale, ranging from “1 – Never” to “5 - A great deal”, with higher scores indicating more contact with people with dementia.
C. The Brief Adolescent Attitudes towards Dementia Scale (Brief A-ADS)(t1, t2) – is a measure consisting of 13 items measuring attitudes towards dementia. The brief version is a refined version of a larger scale designed for use in adolescents [27]. The A-ADS has shown very good internal consistency (α =0.82), and concurrent validity with scores on the Allophilia scale (*r* = 0.72 [28]). The measure includes items such as “every person with dementia has different needs”. It is scored on a 5-point Likert scale, with higher scores indicating more positive attitudes towards dementia.
D. The Kids Insight into Dementia Survey (KIDS) (t1, t2) – a 14-item measure of children’s perceptions of dementia, it captures; ‘personhood’, ‘stigma’ and ‘dementia understanding’ [29]. It has shown very good internal consistency (α = 0.83) and includes items such as “dementia is when something has gone wrong in your brain”. It is scored on a 5 point Likert scale ranging from “5 – agree a lot” to “1 - disagree a lot”, with higher scores indicate increased understanding of dementia.
E. Interest in dementia-related career paths (t1, t2). A single item question was added to explore intention to pursue a career working with people with dementia. “In the future, I would be willing to work with people who have dementia”. No guidance was provided to participants about what type of job roles this encapsulated.
F. Dementia Friends’ satisfaction survey (t2) – researcher-created questionnaire aimed to capture what aspects of the Dementia Friends session adolescents liked and disliked.
G. EmQue-CA (t1) – An 18-item general measure of adolescent empathy, which captures ‘affective empathy’, ‘cognitive empathy’, and ‘intention to comfort’ [30]. The EmQue-CA displayed good internal consistency across each of the subscales (α = 0.70, α = 0.70, and α = 0.74, respectively). Each item has a 3-point Likert response format, where participants rate items “Not True”, “Sometimes True” and “Often True”. Totals were scored according to online syntax [31], in which higher scores reflect higher levels of empathy. Empathy has been suggested to effect changes in attitudes, and therefore we wanted to ensure participants did not significantly differ between groups.
H. A researcher-designed questionnaire to understand what participants within the Dementia Awareness group felt about the initiative (t2). The nine items include statements such as “The dementia awareness class has improved my attitudes towards people living with dementia”, “I now know enough about dementia” and “Overall, I enjoyed the dementia awareness class”. Each item had a five-point response format ranging from “strongly disagree” to “strongly agree”.

### Procedure

Classes (approximately 30 adolescents in each) were assigned to receive Dementia Friends (dementia awareness group) or lessons as usual (control). All adolescents received information sheets about the research, in which they were provided the option to opt-out of the research. Adolescents could choose to participate in the Dementia Friends session without being involved in the research.

All adolescents, irrespective of whether being assigned to the dementia awareness group or control, received the baseline questionnaires at a similar time within each school. These pen and paper questionnaires were completed within normal lesson time, approximately 1 week prior to the Dementia Friends session.

Each Dementia Friends session lasts between 45 and 60 min and, through activities and discussion, covers five key messages that everyone should know about dementia. The five key messages are:

1. Dementia is not a natural part of aging.
2. It is caused by diseases of the brain.
3. It is not just about memory loss.
4. It is possible to live well with dementia.
5. There is more to the person than the dementia.

The session includes question and answers, providing analogies, and interactive tasks. There is no person with dementia or carer voice within Dementia Friends (e.g. videos), with content being presented about people with dementia (e.g. facts and stories about people with dementia). This included getting adolescents to fill in the missing word for the above key messages (referred to as the ‘Broken Sentences’ task) and for them to work in pairs to create a list of steps to make a tasty sandwich (to highlight the importance of cognitive function in everyday tasks, and the implications if there is cognitive impairment). At the end of the Dementia Friends session, adolescents have the opportunity to turn their understanding into action by committing to a dementia-friendly action. At the end of the session, all attendees become a ‘Dementia Friend’ and receive a badge.

The session was run by a volunteer ‘Dementia Champion’ (EJ), who has been trained by Alzheimer’s Society to host such events. EJ has trained 1400 Dementia Friends, has lived experience of caring for someone with dementia, and is a retired social worker.

Following the Dementia Friends session (typically 1 week) all adolescents were provided a follow-up pen and paper questionnaire alongside a debrief sheet.

### Data analysis

Independent and dependent variables, including change scores, were checked for univariate outliers (> 3 * Interquartile Range (IQR)).

Additional steps were taken to control for careless/insufficient effort responses (i.e. responding ‘1’ to all items) by removing them from the analysis. Maximum Long String Index (i.e. maximum number consecutive values) was calculated for items within the KIDS and Brief A-ADS dependent variables. Based on previous recommendations, the item order was randomised prior to calculating [32]. All cases in which the Maximum Longstring Index values that were above 2 SD over the mean were considered as high Insufficient Effort Responders [33], and thus cases were removed.

Categorical variables were recoded into dummy variables (e.g. White British = 1, Non-white British = 0).

Descriptive data (i.e. mean, standard deviation, frequencies) were reported for baseline measures split by group. T-tests and Pearson Chi square tests were used to compare baseline demographics between the control group and dementia awareness group. Fischer’s Exact test was employed in a single comparison, as number of valid cases per group were small (*n* < 5).

A series of Pearson’s correlations were completed between outcome measures (Brief A-ADS and KIDS), to provide evidence of concurrent and test-retest validity.

Univariate analysis (t-test) was initially completed between change scores of attitude domains from the KIDS and Brief A-ADS. A non-parametric test (Mann-Whitney U) was used to compare change scores between groups of the single item about interest in pursuing a dementia-related career path. Effect Size (Cohen’s d) and 95% CIs were also reported.

The KIDS and Brief A-ADS variables were subsequently included for further analysis using a repeated measure ANCOVA. Demographic factors were entered as covariates (i.e. age, ethnicity, gender, direct level of contact), and school of origin were also added as covariates. Effect sizes (Partial η^2^) were reported.

The effect size was broadly interpreted in line with existing guidelines (i.e. small effect, *d =* 0.2) [34], though acknowledge that the benefit of effect sizes is to allow for comparison between outcomes and with other studies [35].

## Results

Three hundred and nineteen adolescents participated in the research and completed both baseline and follow-up assessments. Following the screening of data for outliers and insufficient effort responses, 301 participants remained in the analysis. One hundred and two participants were within the control group and 198 participants were in the dementia awareness group.

On average, participants were 12.6 years old (SD = 0.73), female (*n* = 172, 57.1%) and White British (*n* = 235, 78.1%). There was no significant difference between baseline characteristics between the dementia awareness group and the control group, except for their age (older in the controls; t = 3.04, *p* = 0.003), direct level of contact (higher in dementia awareness group; t = − 1.99, *p* = 0.05), and KIDS total score (higher in dementia awareness group; t = − 2.17, *p* = 0.03). See Table 1 for full descriptive data, split by group.

**Table 1:** Descriptive statistics of baseline characteristics. Statistical comparisons are made between groups

	Control		Dementia Awareness		Statistic	*P* value
	N (%)	Mean (SD)	N (%)	Mean (SD)		
Age		12.7 (0.80)		12.5 (0.67)	t = 3.04	0.003
Gender: Male	44 (43.1%)		82 (40.4%)		χ^2^ = 0.24	0.62
Ethnicity: White British	86 (84.3%)		149 (74.9%)		χ^2^ = 3.51	0.06
Have you heard of dementia or Alzheimer’s before? Yes	101 (99.0%)		190 (95.5%)		–	0.17
EmQue-CA: Affective Empathy		1.18 (0.37)		1.13 (0.40)	t = 0.95	0.34
EmQue-CA: Cognitive Empathy		1.44 (0.40)		1.41 (0.40)	t = 0.64	0.52
EmQue-CA: Prosocial Motivation		1.69 (0.35)		1.60 (0.39)	t = 1.86	0.06
Indirect Contact of Dementia (↑ more contact)		9.20 (4.85)		9.23 (5.26)	t = −0.48	0.96
Direct Contact of Dementia (↑ more contact)		7.38 (2.40)		8.08 (3.00)	t = −1.99	0.05
KIDS Total (↑ better attitudes) (max = 70)		50.63 (8.23)		52.61 (7.02)	t = −2.17	0.03
Brief A-ADS Total (↑ better attitudes) (max = 65)		48.09 (6.46)		48.24 (5.88)	t = −0.20	0.85

*KIDS* Kids Insight into Dementia Survey, *A-ADS* Adolescent Attitudes towards Dementia Scale, *EmQue-CA* Empathy Questionnaire for Children and Adolescents

### Reliability check

The Brief A-ADS and the KIDS total scores had a moderate positive association with each other, both pre and post-test (ps < 0.0001). In addition, pre-test scores (Brief A-ADS and KIDS) of these outcomes had a moderate positive association with post-test scores of the same instruments (ps < 0.0001). See Table 2. Test-retest reliability was checked using the control group data only, the measures displayed questionable reliability for the KIDS (*r* = 0.55, *p* < 0.0001) and acceptable reliability for the Brief A-ADS (*r* = 0.78, *p* < 0.0001).

**Table 2:** Correlations between dementia attitude outcomes at baseline and follow-up

	Brief AADS (T1)	KIDS (T2)	Brief AADS (T2)
KIDS (T1)	0.47 (*p* < 0.0001)	0.60 (*p* < 0.0001)	0.51 (*p* < 0.0001)
Brief AADS (T1)		0.53 (*p* < 0.0001)	0.67 (*p* < 0.0001)
KIDS (T2)			0.67 (*p* < 0.0001)

*T1* Pre-test, *T2* Post-test, *KIDS* Kids Insight into Dementia Survey, *A-ADS* Adolescent Attitudes towards Dementia Scale

### Dementia attitudes

The average KIDS score (t = − 5.57, *p* < 0.0001) significantly improved between pre and post for the dementia awareness group. The Brief A-ADS displayed no statistically significant change between time points (t = 0.29, *p* = 0.77).

When investigating change scores between groups, there was no statistically significant difference between the dementia awareness group and control group on the KIDS and Brief A-ADS outcomes. Effect sizes ranged from non-existent on the KIDS (*d* = − 0.003 *p* = 0.98) to small on the A-ADS (*d* = 0.14, *p* = 0.31). For the full details, please see Table 3.

**Table 3:** Descriptive statistics of dementia attitude scales (pre-test, post-test and change scores). Between group comparisons made between groups on change scores

Change scores	Dementia Awareness			Control			Between group analysis			
	T1 M (SD)	T2 M (SD)	Change M (SD)	T1 M (SD)	T2 M (SD)	Change M (SD)	Effect Size (d)	ES 95% CI	t	p
KIDS total	52.61 (7.02)	55.12 (7.00)	2.45 (6.14)	50.63 (8.23)	52.90 (7.02)	2.47 (7.27)	−0.003	−0.75 – 0.74	0.03	0.98
Brief A-ADS Total	48.24 (5.88)	48.22 (6.56)	−0.12 (5.47)	48.09 (6.46)	47.39 (6.65)	−0.84 (4.11)	0.14	−0.49-0.78	−1.02	0.31

*M* Mean, *SD* Standard Deviation, *T1* Pre-test, *T2* Post-test, *ES* Effect size, *CI* confidence intervals, *KIDS* Kids Insight into Dementia Survey, *A-ADS* Adolescent Attitudes towards Dementia Scale

Within the multivariate models, after factoring covariates, there was no significant main effect of *Time* (F (1,278) = 0.58, *p* = 0.45, η^2^ = 0.002) or *Time x Group (*F (1, 278) = 0.001, *p* = 0.98, η^2^ < 0.0001) for the KIDS outcome. With the Brief A-ADS outcome there was no significant main effect of *Time* (F (1, 238) = 1.23, *p* = 0.27, *η*^*2*^ = 0.005), or *Time x Group* effect (F (1,238) = 1.44, *p* = 0.23, *η*^*2*^ = 0.006).

### Willingness to work with people with dementia

There was no significant difference of change scores between groups on the question about the willingness to work with people with dementia in the future (Z = − 1.29, *p* = 0.20, d = 0.17, 95% CI (0.04–0.30)).

### Reaction to dementia friends

Adolescents that participated in the Dementia Friends were generally satisfied with the session. Data across the session satisfaction questionnaire were positively skewed; the majority of participants enjoyed the session, found it interesting, and subjectively felt it improved both attitudes and knowledge. See Table 4.

**Table 4:** Descriptive data of responses to the satisfaction survey

	MDN (IQR)	Strongly Disagree: 1	Disagree: 2	Neither agree nor disagree: 3	Agree: 4	Strongly Agree: 5	Missing
		N (%)	N (%)	N (%)	N (%)	N (%)	N (%)
The dementia awareness class increased my knowledge of people living with dementia	4 (1)	3 (1.5%)	6 (3.0%)	30 (15.1%)	95 (47.7%)	54 (27.6%)	10 (5.0%)
The dementia awareness class has improved my attitudes towards people living with dementia	4 (1)	0 (0.0%)	8 (4.0%)	61 (30.7%)	87 (43.7%)	31 (15.6%)	12 (6.0%)
The dementia awareness class was interesting.	4 (1)	1 (0.5%)	8 (4.0%)	40 (20.1%)	102 (51.3%)	35 (17.6%)	13 (6.5%)
The dementia awareness class was too long.	3 (1)	15 (7.5%)	68 (34.2%)	82 (41.2%)	19 (9.5%)	4 (2.0%)	11 (5.5%)
I am now more confused about dementia	2 (1)	57 (28.6%)	85 (42.7%)	37 (18.6%)	7 (3.5%)	2 (1.0%)	11 (5.5%)
I would have liked to have met a person living with dementia during the class	4 (1)	7 (3.5%)	14 (7.0%)	68 (34.2%)	61 (30.7%)	38 (19.1%)	11 (5.5%)
I now feel more comfortable talking about dementia	4 (1)	2 (1.0%)	6 (3.0%)	73 (36.7%)	79 (39.7%)	29 (14.6%)	10 (5.0%)
I now know enough about dementia	4 (1)	7 (3.5%)	15 (7.5%)	62 (31.2%)	80 (40.2%)	24 (12.1%)	11 (5.5%)
Overall, I enjoyed the dementia awareness class	4 (1)	2 (1.0%)	5 (2.5%)	43 (21.6%)	98 (49.2%)	41 (20.6%)	10 (5.0%)

*IQR* Interquartile range, *MDN* Median

## Discussion

The present study set out to explore the effects of a one-off dementia awareness session (Dementia Friends) on attitudes of dementia within adolescents. Improved attitudes toward dementia and reduced stigma are often cited as the key outcome of interest in these initiatives, though they generally have not been thoroughly evaluated, particularly using validated instruments.

Univariate analysis revealed that whilst there was an improvement in one outcome (KIDS) in the dementia awareness group, this change did not significantly differ to those in the control group. There was however, a small positive effect on the Brief A-ADS change scores between the awareness group and the control group. However, this difference was non-significant and driven in part by a very small decline in attitudes in the control group, and a very small improvement in the awareness group. Comparing these effect sizes to the existing literature is difficult, primarily due to the lack of evidence, though in comparing it to the wider mental illness stigma literature, the effect sizes reported here are much smaller (*d* = 0.45) [14]. It should be noted that the 95% CIs of the effect sizes reported in this study were particularly wide which indicates a level of imprecision or that the sample size is possibly too small [36, 37], therefore requiring replication in a larger sample before firm conclusions can be drawn.

It should be highlighted that the change scores remained small on average across all groups, indicating that even if there were benefits to attitudes towards dementia, there needs to be a dialogue about whether this change is enough to be considered meaningful. Typically, attitude change is meant to act as an intermediate for behavioural change, though the reasonably short follow-up period prevented such data from being captured. However, items related to behavioural intention are included within the questionnaires (e.g. "If I saw someone with dementia struggling to do something, I would help them"), which may predict to some extent eventual behaviour [38]. We also included a single item question about the willingness to work with people with dementia, to acknowledge the need to recruit and retain social care workers and nurses [39, 40], who will support the increasing number people living with dementia in the UK. Within this behavioural intention item, there was no significant differences between groups in terms of change scores.

Adolescents’ positive reaction to the Dementia Friends session, reflect those reported within adolescent focus group discussions of an overlapping sample (Unpublished data). It is important to recognise that discrepancies between objective and self-reported gains is well documented [41, 42], with the latter being subject to social desirability bias and halo error [43]. This means that the data on self-reported gains in attitudes should be interpreted with some caution because the respondents may, consciously or unconsciously, have been seeking to give the “right” answer rather than one that reflected their actual perceptions.

Due to the novelty of this research, there are a limited number of validated measures that capture attitudes towards dementia in young people. The Brief-A-ADS and KIDS questionnaires are relatively new measures, though both have indicated good internal consistency (Brief A-ADS from unpublished data) [29]. In addition, the KIDS (*r* = 0.76) has good external validity against an established dementia attitudes question for adults (Dementia Attitudes Scale; [44]), whilst the Brief A-ADS is strongly associated with both the 23-item A-ADS (*r* = 0.95) and Allophila scale (*r* = 0.72) (Unpublished data). In the current study, the KIDS and Brief A-ADS had moderate associations with each other (*r* = 0.47–0.67), which indicates that they broadly measure associated constructs. However, it is important to recognise that these questionnaires do measure different underlying factors, which could explain variations in effects reported here. In addition, whilst at face level the KIDS questionnaire does not appear to have any culturally specific items related to dementia, the original questionnaire was developed with a slightly younger (age 9–12) Australian sample, which could subsequently impact its validity in the current study within a different country. Notably the test-retest reliability was questionable for the KIDS in the current sample. Irrespective, it is important to consider that these measures capture explicit attitudes only, and are subject to social desirability bias, therefore may underestimate actual stigma [45].

It is important to consider the sample characteristics, and how they reflect adolescents more broadly, as this could affect the external validity of the study. Within the current data set, 4.5% of adolescents had not heard of *dementia* or *Alzheimer’s disease*. This appears to be substantially lower than that reported in Sydney Australia, in which 34.5% had not heard of these terms [29]. This could be explained by cultural and educational differences, or due to the sample from Baker and colleagues being a slightly younger age group (M = 10.5, SD = 0.62). The current sample’s underlying knowledge of dementia is however, similar to that of a cohort of adolescents within the South East of England that were asked whether they had heard of dementia (4.3% had not heard of the term *dementia*) [46]. Irrespective of variations of initial knowledge of dementia, the attitude scores reported were largely in line with those reported both in Australia based on the KIDS (M = 50.48, SD = 7.51) [29], and within England using the Brief A-ADS (*n* = 630, M = 47.91, SD = 6.59). Therefore, we can assume that our sample has similar baseline attitudes and knowledge of dementia compared to other adolescents reported elsewhere. It is important to highlight the findings reported here may not be replicated in other countries or demographics (e.g. age, ethnicity). In addition, we did not capture response rates, and therefore are unable to describe the extent to which there is a non-response bias in our data. Inherently, we would envisage that those who participated in the research are likely to represent individuals who were most engaged with topic, and hence satisfaction (in particular) may have been higher in our sample.

Looking to the future, it is important to consider whether the Dementia Friends model is the most appropriate means of attitude change in adolescents, especially considering there was little effect on attitudes within one month of the Dementia Friends session. It is important that similar discussions about Dementia Friends are had in adult samples, particularly in consideration that young people may be more susceptible to attitude change [47, 48] and different anti-stigma strategies are more effective depending on age [14]. In addition, there are minor variations in terms of tasks and content covered in Dementia Friends sessions depending on the groups age, though there needs to be a discussion of whether a different approach is needed for young people, if Dementia Friends is effective in changing attitudes in adults (e.g. [23]). This is not to say that Dementia Friends should be completely discarded, not least because adolescents tended to enjoy the session, and subjectively believed it improved their attitudes and knowledge. In addition, it did not negatively affect attitudes towards dementia. Importantly, the feasibility of Dementia Friends (i.e. single session, run by a volunteer), makes it an attractive model for resource and time-limited schools. Such considerations are important because schools might struggle to prioritise dementia as a topic, or have it feature heavily within the curriculum. If schools are happy to do so, the Alzheimer’s Society do have resources to achieve a more comprehensive education programme [49], though again there is limited transparency on its effectiveness in changing attitudes towards dementia.

## Conclusions

Dementia Friends has been a success in terms of numbers of people enrolled, however, as stated previously, achieving a set number of Dementia Friends does not “*guarantee the fight against stigma, prejudice and discrimination will have been won*” [50] (para. 1), and therefore there is a need to better understand the impact of these initiatives [51]. Overall, it is clear that Dementia Friends is a well-received initiative in adolescents that does not have a negative effect on their attitudes towards dementia in the short-term, though there is a need for further replication in larger randomised controlled trials.

## Data Availability

The datasets generated during and/or analysed during the current study are not publicly available due ethical restrictions but are available from the corresponding author on reasonable request.

## Abbreviations

A-ADS: Adolescent Attitudes towards Dementia Scale
ALoCDs: Adolescent Level of Contact with Dementia Scale
CI: Confidence Interval
IQR: Interquartile Range
KIDS: Kids Insight into Dementia Survey
M: Mean
MDN: Median
SD: Standard Deviation

## Footnotes

1 An academy sponsor led school, is one where an individual or organisation that has received approval from the Department for Education to support the academy. An academy is funded by the Department for Education, but not independent of local authority control.

2 The Progress 8 scores represents how much progress pupils at this school made between the end of primary school (typically aged 11) and the end of secondary school (typically aged 16), compared to pupils across England who achieved similar results at the end of primary school. A score above zero means pupils made more progress on average than pupils across England who got similar results at the end of primary school. A score below zero means pupils made less progress on average than pupils across England who got similar results at the end of primary school.
